# Transfer of Energy Capacitive and Resistive Therapy Versus Dry Needling for Active Upper Trapezius Myofascial Trigger Points: Effects on Pain and Cervical Range of Motion a Randomized Controlled Trial

**DOI:** 10.3390/healthcare14070860

**Published:** 2026-03-27

**Authors:** Tomasz Piętka, Katarzyna Knapik, Grzegorz Onik, Karolina Sieroń

**Affiliations:** 1 Department of Physical Medicine, Faculty of Health Sciences in Katowice, Medical University of Silesia in Katowice, 40-055 Katowice, Poland; 2 Institute of Medical Sciences, Department of Internal Medicine and Gastroenterology, WSB University, 41-300 Dąbrowa Górnicza, Poland

**Keywords:** myofascial trigger point, myofascial pain syndrome, TECAR therapy, dry needling, upper trapezius, pain, range of motion, muscle strength

## Abstract

**Background and Objectives**: This study aimed to evaluate the effectiveness of Transfer of Energy Capacitive and Resistive (TECAR) therapy in treating active myofascial trigger points (MTrPs) in the upper trapezius muscle (UT) and to compare it with the effects of dry needling (DN). **Materials and Methods**: We recruited 29 men (mean age: 35.52 ± 5.73 years) with active MTrPs in the UT. Participants were randomly assigned to two groups: TECAR (n = 17) and DN (n = 12). Treatment was administered twice, with a 7-day interval between sessions. PPT, pain intensity (NRS), UT muscle strength (dynamometer), and cervical spine range of motion (ROM) were measured before treatment, immediately after each therapy session, and at a 30-day follow-up. Data were analyzed using parametric or non-parametric tests depending on data distribution (*p* < 0.05). **Results**: Both groups showed significant increases in PPT, but TECAR reduced NRS significantly more than DN (*p* < 0.001), demonstrating superior immediate analgesia. While TECAR temporarily decreased unaffected UT strength, it provided broader improvements in cervical mobility (flexion: 19.5%, contralateral rotation: 13.1%). Over 30 days, both groups improved PPT (TECAR: ~110%; DN: ~63%) and NRS (TECAR: ~97.1%; DN: ~84.5%). The TECAR group consistently outperformed DN in long-term pain reduction and achieved more substantial improvements in ROM. **Conclusions**: TECAR therapy appears to provide immediate and longer-term analgesic effects in the treatment of active MTrPs in the UT, although its impact on cervical ROM seems relatively limited compared with DN. It may therefore represent a useful, though less commonly applied, option for MTrPs management.

## 1. Introduction

Myofascial pain syndrome (MPS), caused by myofascial trigger points (MTrPs), affects up to 85% of individuals presenting with chronic pain in clinical settings [1], with the highest incidence in people aged 27–50 years [2]. It has been noted that 21% of people seek care at orthopedic clinics due to MPS, and 85–93% of patients attend pain management centers [3]. MPS reduces the quality of individuals’ lives and impairs both physical and social functioning. Moreover, it poses an economic burden on the health care system [4]. Repetitive and/or precise movements as well as lifting heavy objects can cause micro-injuries that may lead to MTrPs [5]. They are defined as hyperirritable spots within taut bands of skeletal muscle fibers that generate pain [6]. Based on clinical presentation, MTrPs can be classified as either active or latent. Active MTrPs cause spontaneous local and referred pain at rest, whereas latent MTrPs produce pain only in response to palpation or stretching [7].

The upper trapezius (UT) is the most common location for MTrPs [8,9,10]. It is estimated that 78.8% of healthy individuals have latent MTrPs in this region [11]. In contrast, among individuals with MPS, the presence of MTrPs in UT has been confirmed in 93.75% of cases [12]. Clinically, MTrPs in the UT may manifest as increased muscle tone, local pain, tension-type headaches, neck pain, vertigo, limited cervical range of motion, and disturbances in the scapulohumeral rhythm [8]. Additionally, active MTrPs in the UT contribute to mechanical neck pain by facilitating arthrogenic muscle inhibition, a physiological process where nociceptive input reflexively impairs motor unit recruitment and synchronization [13]. This resultant muscle weakness is characterized by reduced peak force production and accelerated metabolic fatigue, which compromises cervical motor control and perpetuates chronic musculoskeletal dysfunction [14].

The pathophysiology of MTrPs is centered on the integrated hypothesis of an “energy crisis,” where the excessive release of acetylcholine at the motor endplate induces sustained sarcomere contraction, leading to local microvascular compression and ischemia [13]. This metabolic distress triggers the accumulation of pro-inflammatory mediators (e.g., Substance P, bradykinin) and a decrease in pH, which sensitizes local nociceptors and results in the characteristic formation of a palpable taut band and localized pain [15].

Physiotherapists use various methods to treat MTrPs in the UT, including massage, stretching, manual therapy, and the spray-and-stretch technique [7,16,17]. To date, dry needling (DN) has also been shown to be effective in the treatment of MTrPs, with its efficacy comparable to that of standard treatments [8,9,18,19,20]. DN improves local blood flow [21], provides analgesic effects [22], and promotes sarcomere relaxation [23], making it a potentially effective intervention for MTrPs. Considering the physiological effects of capacitive and resistive energy transfer (TECAR) therapy, it may also be a viable treatment option for MTrPs [24]. TECAR therapy is a non-invasive modality that uses high-frequency current (300 kHz–1 MHz) to treat musculoskeletal disorders [25]. According to Yeste-Fabregat et al. [24] and Clijsen et al. [26], TECAR therapy generates heat within tissues, leading to temperature-dependent biological effects [27]. Bito et al. [28] reported that TECAR therapy improves blood circulation in tendons and increases hemoglobin saturation. Additionally, it reduces muscle tension, enhances lymphatic drainage [29], alleviates pain [30], increases connective tissue elasticity [31], stimulates enzymatic activity, and improves nerve conduction [26]. For these reasons, TECAR therapy is recommended for various medical conditions, including back pain syndrome [30,31], neuralgia, edema, tendinopathy, muscle cramps [32], degenerative joint disease [33,34], and plantar fasciitis [35].

Considering the wide range of biological effects mediated by TECAR therapy and the limited number of studies investigating its application in the treatment of active MTrPs, the present study aims to address this gap in the literature. DN, with its well-documented effectiveness, may serve as a valuable reference for evaluating the clinical utility and efficacy of TECAR therapy in the management of active MTrPs.

Therefore, the primary study aim was to evaluate the effectiveness of TECAR therapy in the treatment of active MTrPs within UT. The secondary study aim was to compare the effectiveness of TECAR therapy versus DN in the treatment of active MTrPs, specifically evaluating their impact on the Pressure Pain Threshold (PPT), cervical range of motion (ROM), and the muscle strength of the UT.

## 2. Materials and Methods

### 2.1. Trial Registration

This trial was prospectively registered at ClinicalTrials.gov with identifier NCT06273514 (Comparison the Effects of TECAR with Dry needling in the treatment of Myofascial Trigger Points). The study was conducted as an interventional, parallel-group, randomized controlled trial (RCT) with no masking (open-label).

### 2.2. Characteristics of the Study Group

**Figure 1:**
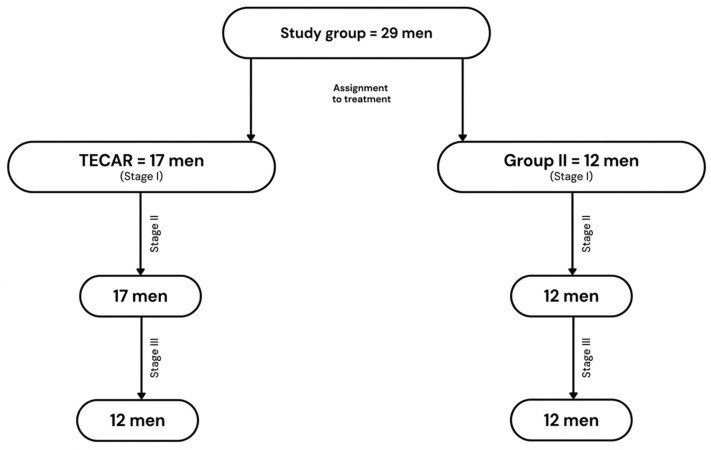
Assignment to treatment groups and the number of participants at each stage of the study.

Individuals with mechanical neck pain were recruited from patients seeking care at a physiotherapy clinic in Ruda Śląska, Poland. The participants were recruited between 1 December 2020 and 31 December 2022. Ultimately, 29 men aged 27 to 45 years (mean age: 35.52 years ± 5.73 years) with active MTrPs in the UT were included in the study. The number of participants at each stage of the study is presented in Figure 1.

The following exclusion criteria were set: musculoskeletal pain syndrome, MTrP therapy in the UT region during the last year; history of neck trauma, cervical spine and humeral joint surgeries; cervical radiculopathy, chronic and acute diseases of the cardiovascular, respiratory, and nervous systems; endocrine disturbances; skin lesions; acute inflammation; cancer; and BMI > 30 kg/m^2^. The criteria that had to be met to diagnose active MTrP were in accordance with the Delphi panel consensus [36]. Therefore, to discover active MTrPs, the following symptoms had to occur during functional examination: spontaneous pain at rest, a palpable tender spot in the taut band with reproduction of the symptoms and local twitch response. In both groups, active MTrPs were located dominantly in the right UT. In the TECAR group, MTrP was located on the right side in 64.7% of participants (n = 11), while in the DN group, it was 75% (n = 9). Notably, participants did not ingest any analgesic drugs during the entire observation period. The characteristics of the study groups due to conducted treatment are presented in Table 1.

Considering the significant difference in body weight between the groups, a regression model was built to examine the association between body weight and baseline measurements. However, none of these associations were statistically significant (PPT: β = −0.01, *p* = 0.09, R^2^ = 0.17; muscle strength on the affected side: β = 0.07, *p* = 0.78, R^2^ = 0.02; muscle strength on the unaffected side: β = 0.06, *p* = 0.54, R^2^ = 0.05).

### 2.3. Randomization

Participants were randomly assigned to one of two treatment groups: TECAR or DN, using a simple coin flip method. Upon enrollment and after providing informed consent, each participant was assigned a unique identification number. The treatment allocation for each participant was determined by the outcome of a single coin toss. For each participant, ‘heads’ was pre-determined to correspond to the TECAR group, and ‘tails’ was pre-determined to correspond to the DN group. The coin was flipped by the principal investigator, out of sight of the participant to ensure impartiality. The outcome of the coin flip was recorded alongside the participant’s identification number and treatment allocation. This process continued until all 29 participants were assigned to a treatment group, resulting in 17 participants in the TECAR group and 12 participants in the DN group. While this method aims for equal probability of assignment, the resulting group sizes may differ slightly due to chance, as observed in this study.

### 2.4. Intervention

The study protocol was approved by the Bioethics Committee of the Medical University of Silesia in Katowice (resolution number: PCN/0022/KB1/37/I/20). All procedures were conducted in accordance with the Declaration of Helsinki. Written informed consent was obtained from all participants prior to their involvement in the study. The study adhered to the CONSORT reporting guidelines. Each participant completed a treatment regimen consisting of two sessions, with a one-week interval between them. Diagnostic and therapeutic procedures were performed by a well-trained and experienced physiotherapist.

#### 2.4.1. Dry Needling

**Figure 2:**
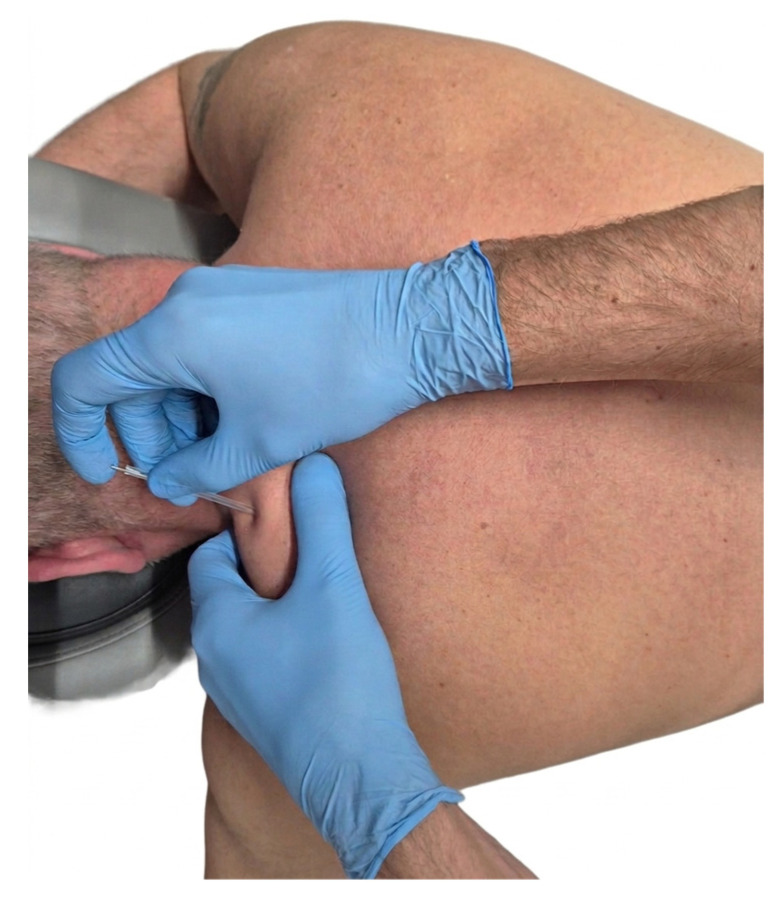
DN procedure, illustrating needle insertion and therapist’s hand positioning for tissue stabilization.

DN was performed using SOMA needles (25 mm × 0.3 mm). During the procedure, the participant was positioned in the prone position. The affected region of the UT was disinfected prior to needling. The taut band was identified between the researcher’s thumb and index finger. The needle, contained within a plastic guide tube, was placed over the MTrP in the UT and then directly inserted into the MTrP. The presence of a local twitch response confirmed the accuracy of the needle placement [37]. Needling was carried out using Hong’s “fast in, fast out” technique [3]. The procedure and clinical application of the DN technique are illustrated in Figure 2.

#### 2.4.2. Transfer of Energy Capacitive and Resistive Therapy

**Figure 3:**
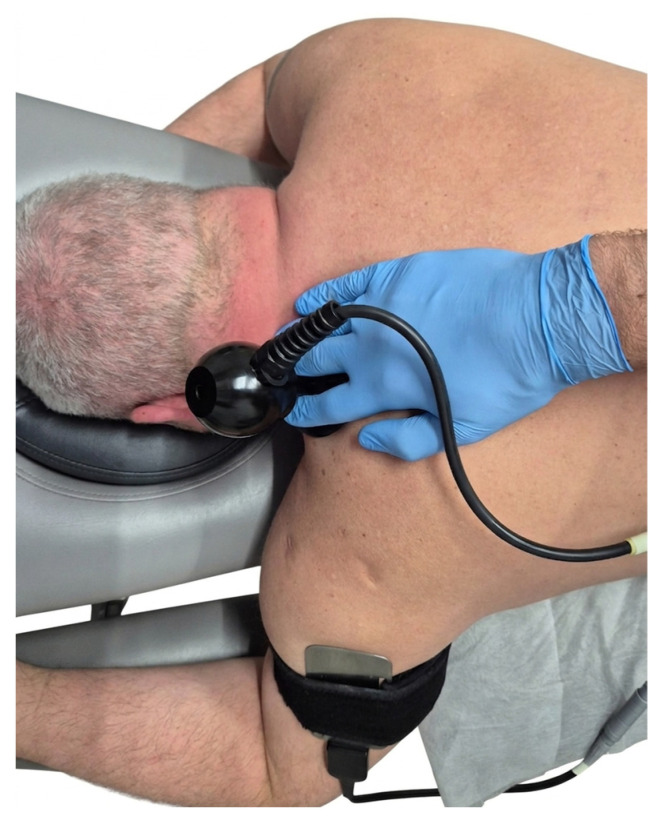
TECAR therapy procedure, illustrating capacitive (CET) and passive electrodes placement and therapist’s hand positioning for dynamic application.

TECAR therapy was performed using a WINBACK^®^ 1s device (Daeyang Medical Co. Ltd., Wonju-si, Gangwon-do, Republic of Korea), with a maximum output of 100 W. A 500 kHz frequency capacitive electrode was applied directly to the active MTrP in the UT, while a closed electrode was placed distally on the arm. Low-pulse modulation was used during treatment, with half-second pauses, allowing the current to be directed specifically into the MTrP without affecting the entire muscle. Each exposure lasted 10 min. The current dose, which was participant-dependent, ranged from 40 to 60% of the maximum output (100% = 100 Watts). No side effects or adverse reactions were observed in any of the participants. The placement of electrodes and the treatment procedure for TECAR therapy are illustrated in Figure 3.

### 2.5. Assessment

Each participant underwent three assessments. The first and second measurements were taken during the therapy sessions (before and after the intervention). The third measurement was conducted within 30 days of the first therapeutic session to evaluate whether the treatment had a lasting effect. To maintain consistency across all clinical parameters, measurements for PPT, NRS, muscle strength, and ROM were performed at four standardized intervals: baseline (T0), immediately following the first session (T1), before second session (T2), immediately following the second session (T3), and at a 30-day follow-up (T4). Specifically, T0 and T1 represent Stage I of the study, while T2 and T3 constitute Stage II.

#### 2.5.1. Pain Measurement

**Figure 4:**
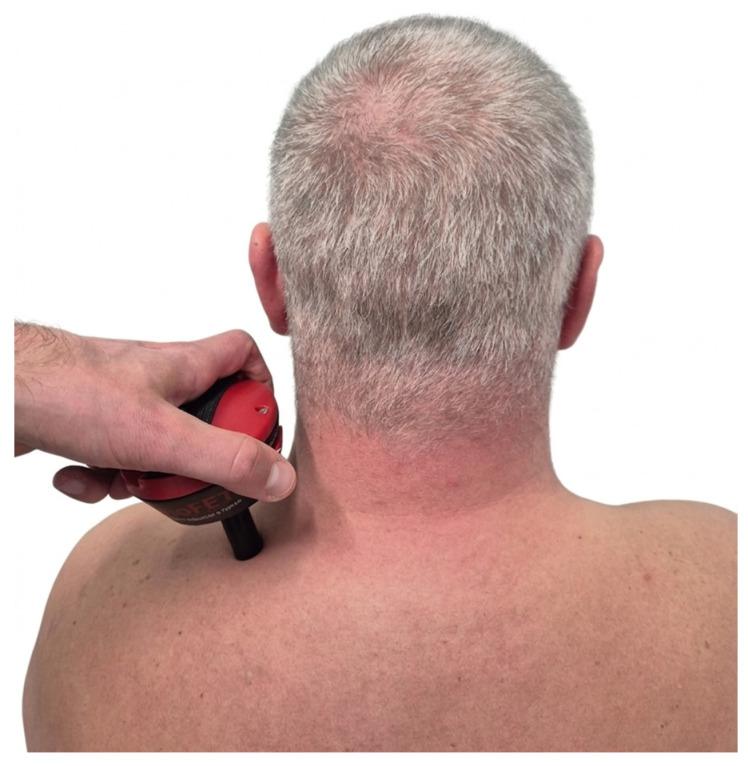
PPT assessment, illustrating the algometer placement and the therapist’s hand positioning.

Pressure pain threshold (PPT), defined as the minimum force applied which induces pain, was assessed using an algometer BioFEET V3 (MusTec, Almere, The Netherlands). The head of the algometer was placed on the MTrP in the UT and then pressed until the participant verbally expressed their PPT. The measurements were performed three times with a 30 s break. The results are presented as the mean value of these measurements and are expressed in kg/cm^2^. PPT is considered a reliable and valid method for the quantitative assessment of myofascial pain. Studies report excellent intra-rater and inter-rater reliability (ICC = 0.91–0.98) specifically for the UT in symptomatic populations [38]. The application of the algometer during PPT measurement is shown in Figure 4.

Moreover, pain intensity was also assessed using a numerical rating scale (NRS). This pain screening tool uses a 0–10 scale, with 0 indicating no pain and 10 indicating the worst imaginable pain. NRS is a widely validated tool for assessing pain intensity in musculoskeletal disorders. It has shown excellent test–retest reliability (ICC = 0.67–0.96) and high construct validity when compared to the Visual Analogue Scale [39].

#### 2.5.2. Muscle Strength Measurement

**Figure 5:**
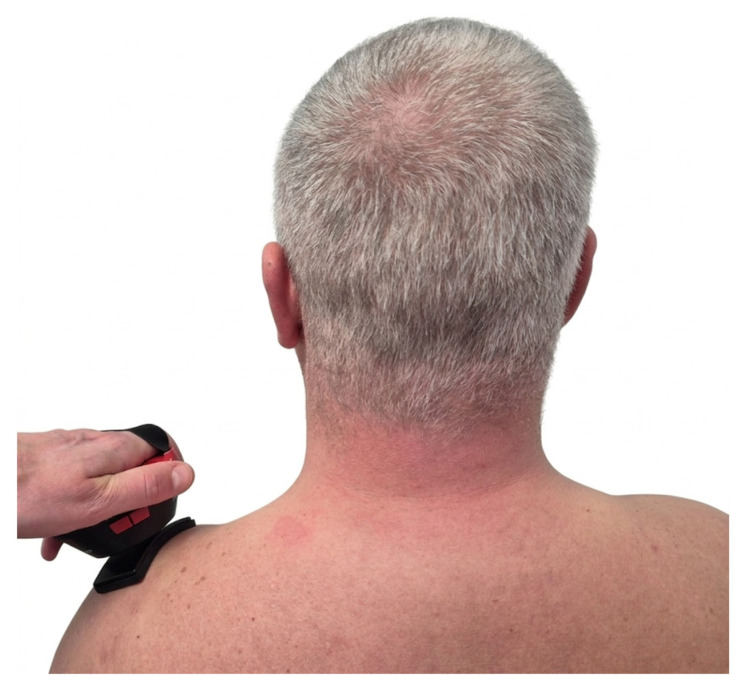
Muscle strength assessment using a hand-held dynamometer, illustrating the device stabilization technique and the therapist’s hand positioning.

Muscle strength was measured using a dynamometer (BioFEET V3; -MusTec, Muscle Dynamic Technology BV, Louis Chrispijnstraat1, The Netherlands 1325 PC ALMERE). We assumed that scapula elevation was the indicator of movement for the UT. During the assessment, the humeral joint remained in a natural position with proximal stabilization. The head of the dynamometer was then placed on the acromion. The physiotherapist resisted active movements to provoke isometric contraction of the UT. Measurements were performed thrice on both sides of the body. The patient’s positioning and the application of the hand-held dynamometer are presented in Figure 5. To ensure measurement reliability and account for the learning effect, three consecutive trials were performed on each side of the body. The peak value (Maximum Voluntary Contraction, MVC) was selected for statistical analysis, as the highest result typically represents the true physiological limit of the muscle and minimizes the confounding influence of submaximal effort or early-onset fatigue often reflected in averaged data. The highest result was obtained for statistical analysis and was expressed as kG. Handheld dynamometry has been validated as a reliable alternative to isokinetic dynamometry for measuring MVC. It demonstrates good concurrent validity (r = 0.86) and excellent intra-trial reliability (ICC > 0.96) for scapular elevation [40].

#### 2.5.3. Range of Motion Measurement

**Figure 6:**
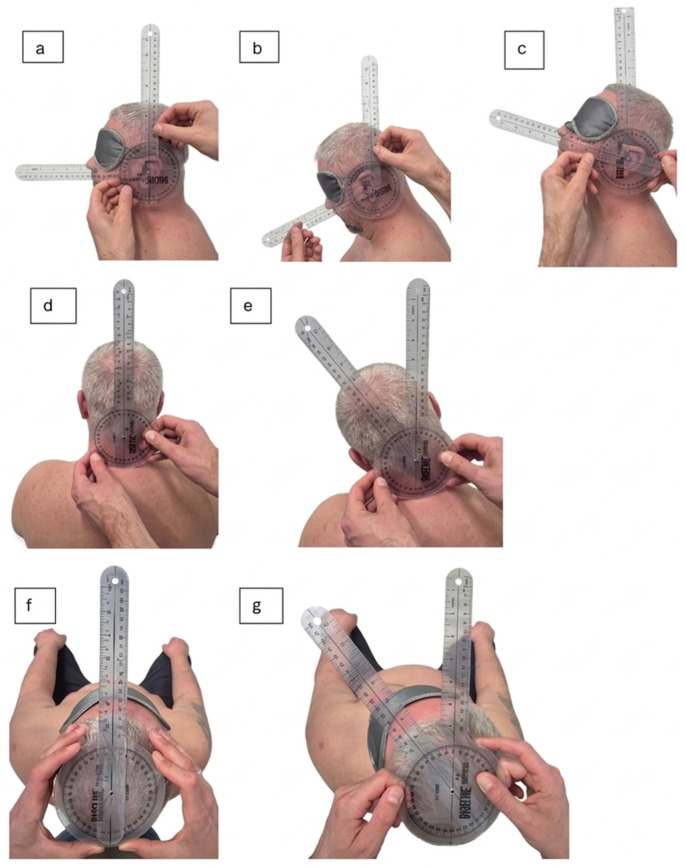
ROM assessment using a manual goniometer, illustrating the alignment of the goniometer’s arms with anatomical landmarks and the therapist’s hand positioning. (**a**) Flexion/Extension starting position, (**b**) Flexion ending position, (**c**) Extension ending position, (**d**) Side bending starting position, (**e**) Side bending ending position, (**f**) Rotation starting position, (**g**) Rotation ending position.

Cervical spine ROM was measured using a goniometer (BASEline, Fabrication Enterprises, Inc. Elmsford, NY 10523, USA). The following movements were assessed: flexion, extension, rotation, and side bending. Each movement was performed three times. The results are presented as the mean value of these measurements and are expressed in degrees (°). During the measurements, the participants sat. While measuring cervical spine flexion and extension, the axis of the goniometer was placed on the auricle opening. During the rotation, the axis of the goniometer was placed at the top of the skull. Side bends were measured with the goniometer axis placed on the spinal process of the axis (C2). The manual goniometric measurement procedure is visualized in Figure 6. While digital tools exist, the universal manual goniometer remains a clinically valid and reliable instrument when used by a single experienced examiner. Recent comparative studies demonstrate that manual goniometry provides consistent and reproducible data for cervical flexion, extension, and lateral flexion (ICC > 0.80) [41].

### 2.6. Statistical Analysis

Statistical analysis was performed using STATISTICA 13.3 PL software. Quantitative variables are presented as means (M) and standard deviations (SD), while qualitative variables are presented as percentages. The normality of data distribution was assessed using the Shapiro–Wilk test. Intragroup comparisons were performed using the Wilcoxon signed-rank test and Student’s *t*-test. Intergroup comparisons were conducted using the Mann–Whitney U test. The chi-square (χ^2^) test was applied to compare qualitative variables. The homogeneity of variances was tested using Levene’s test. If variances were not homogeneous, the Kruskal–Wallis one-way analysis of variance by ranks with a post hoc test was used for intragroup comparisons. If variances were homogeneous, ANOVA with Tukey’s post hoc test was applied. A linear regression model was used to verify the relationship between body weight and baseline results in participants. The level of statistical significance was set at *p* < 0.05. Deltas (∆) were calculated to assess the magnitude of changes in specific variables.

Considering previous reports, we assumed a large effect size of 0.8. With an α error probability of 0.05, sample sizes of 17 and 12 for groups 1 and 2, respectively, yielded a statistical power of 0.66.

## 3. Results

### 3.1. Pain Intensity and Pressure Pain Threshold

In men assigned to the TECAR group, treatment led to a significant increase in the PPT at T1 (approximately 45.55%) and T3 (approximately 15.9%). In contrast, in males treated with DN, the PPT increased only at T1 (approximately 24.7%). However, the magnitude of the changes did not differ significantly between the groups. Subjective pain assessment using the NRS revealed a significant decrease in pain intensity at T1 in both groups. In the TECAR group, pain intensity decreased by approximately 69.24%, while in the DN group, it decreased by approximately 18.94%. At T3, a statistically significant analgesic effect was observed only in the TECAR group (a decrease of about 77.54%). Furthermore, analysis of the magnitude of changes in pain intensity revealed that the changes at both T1 and T3 were significantly greater in the TECAR group (Table 2).

### 3.2. Muscle Strength

At T1, the application of both TECAR and DN resulted in a non-significant decrease in muscle strength in the affected UT. Notably, a statistically significant reduction in muscle strength was observed in the unaffected UT within the TECAR group following the first therapeutic session (approximately 10.35%). Furthermore, the change in muscle strength (∆) in the unaffected UT during T1 was significantly different between the DN and TECAR groups. During the T3, minor, statistically non-significant alterations in muscle strength in the affected UT were noted in both treatment groups, and no significant differences in ∆ muscle strength were observed between the groups (Table 3).

### 3.3. Range of Motion

At T1, the TECAR group experienced statistically significant improvements in several cervical spine movement directions. Specifically, cervical spine extension increased by approximately 13.6%, flexion increased significantly by 19.5%, and rotation contralateral to the MTrP showed a significant increase of 13.1%. Furthermore, contralateral side bending also improved significantly, by 23.1%. During T3 of TECAR treatment, significant improvements were observed in homolateral side bending (increase of 15.0%), contralateral side bending (increase of 12.2%), cervical spine extension (increase of 5.4%), and homolateral rotation (increase of 6.8%). At T3 of DN treatment resulted in statistically significant increases in cervical spine extension (approximately 15.1%), homolateral side bending (16.5%), and contralateral side bending (17.1%). Additionally, the DN group showed a trend toward improvement in homolateral rotation (10.5%) and a significant improvement in contralateral rotation (increase of 11.0%). At T3, the DN group did not demonstrate any statistically significant changes in any of the measured cervical spine ROM. The comparative analysis of changes ∆ between the groups revealed significant differences only in the side bending homolateral to affected UT. The DN group showed an approximate decrease of 3.16%, while the TECAR group exhibited an approximate increase of 14.99% in the measured ROM (Table 4).

### 3.4. Follow-Up Results

To compare results between groups, pre-therapy (T0 and T2) values and T4 measurements were analyzed. Statistical analysis revealed significant differences across all observational stages in both the TECAR and DN groups. In the TECAR group, PPT increased non-significantly by approximately 41% from T0 to T2 (*p* = 0.053), and significantly by approximately 49% from T2 to T4 (*p* = 0.002), resulting in an overall highly significant increase of approximately 110% from T2 to T4 (*p* < 0.0001).

**Figure 7:**
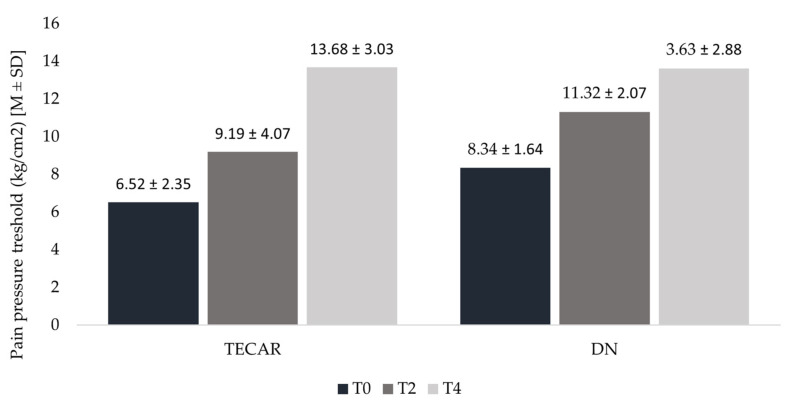
PPT in consecutive measurements in TECAR and DN groups. Legend: T0—baseline measurement; T2—measurement before second session; T4—follow—up after 30 days of first treatment.

Conversely, the DN group demonstrated a significant PPT increase of approximately 36% from T0 to T2 (*p* = 0.007), and a further significant increase of approximately 20% from T2 to T4 (*p* = 0.04), leading to a total highly significant increase of approximately 63% from T0 to T4 (*p* < 0.0001) (Figure 7).

**Figure 8:**
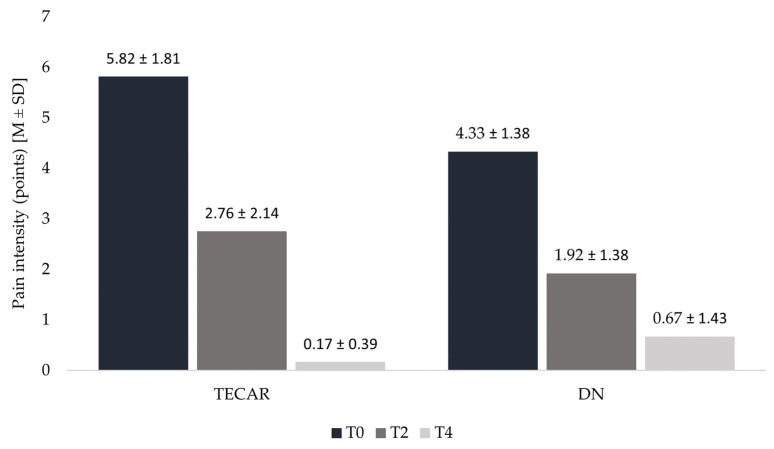
Pain intensity assessed with NRS in consecutive measurements in TECAR and DN groups. Legend: T0—baseline measurement; T2—measurement before second session; T4—follow—up after 30 days of first treatment.

Significant 53. decrease in NRS scores occurred from T0 to T2 (*p* = 0.008), followed by a further highly significant 93.8% decrease from T2 to T4 (*p* = 0.02), resulting in an overall highly significant 97.1% reduction from T0 to T4 (*p* < 0.0001). Notably, no patient in the TECAR group reported pain at follow-up. The DN group also experienced a statistically significant reduction in pain, although to a lesser extent. A significant 55.8% reduction was recorded from T0 to T2 (*p* = 0.045), followed by a significant 65.1% reduction from T2 to T4 (*p* = 0.026), resulting in an overall highly significant 84.5% reduction from T0 to T4 (*p* < 0.0001) (Figure 8).

**Figure 9:**
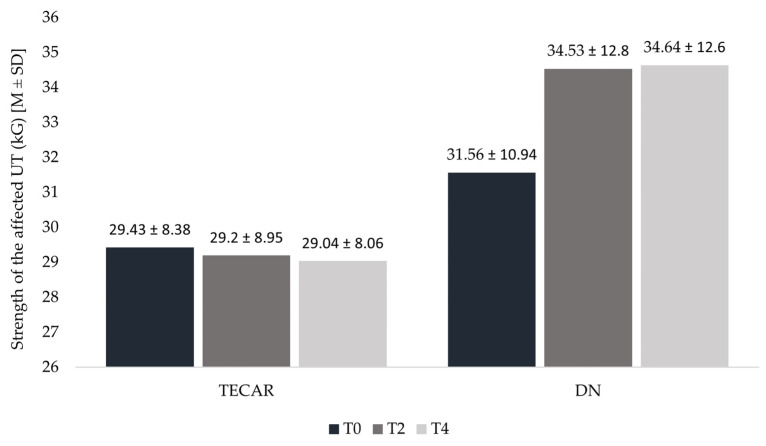
Muscle strength of the affected UT in consecutive measurements in TECAR and DN groups. Legend: T0—baseline measurement; T2—measurement before second session; T4—follow—up after 30 days of first treatment.

**Figure 10:**
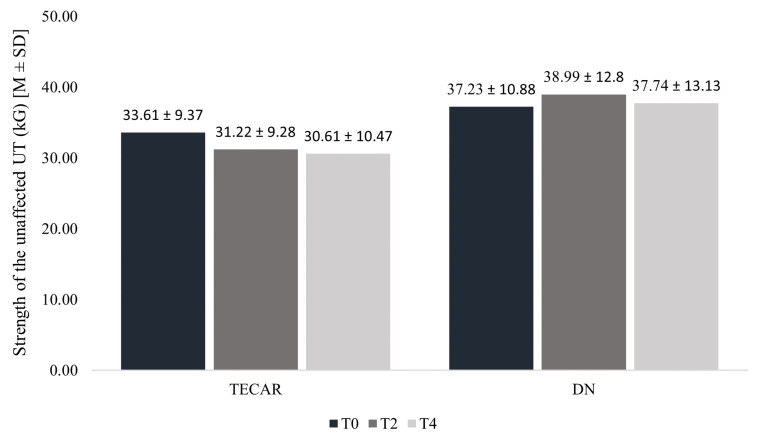
Muscle strength of the unaffected UT in consecutive measurements in TECAR and DN groups. Legend: T0—baseline measurement; T2—measurement before second session; T4—follow—up after 30 days of first treatment.

Although the strength of the affected UT in males assigned to the DN group showed a consecutive increase over the course of the study, these changes were not statistically significant. In the TECAR group, a decrease in affected UT muscle strength was observed during T4 compared to T0 and T2; however, this change was also statistically insignificant (Figure 9). Additionally, in both the DN and TECAR groups, the muscle strength of the unaffected UT remained stable across all time points, with no significant differences observed (Figure 10).

Consecutive measurements of cervical spine ROM revealed significant changes in the TECAR group for flexion, extension, side bending contralateral to the affected UT, rotation homolateral and contralateral to the affected UT. Specifically, flexion increased significantly over time (*p* = 0.048), with post hoc analysis indicating a significantly greater ROM at T4 compared to T0 (approximately 21.1%). In the DN group, a significant increase in extension was observed (*p* = 0.007), with post hoc analysis revealing significantly greater extension at both T2 and T4 compared to T0 (approximately 23.3% and 24.4%, respectively). Side bending homolateral to the affected UT did not demonstrate significant changes in either group (DN: *p* = 0.21; TECAR: *p* = 0.33). However, side bending contralateral to the affected UT significantly increased in both groups. In the DN group, post hoc comparisons showed significant improvements from T0 to T2 (*p* < 0.03) and from T0 to T4 (*p* < 0.006), corresponding to increases of approximately 13.4% and 17.1%, respectively. In the TECAR group (*p* = 0.01), post hoc analysis showed significant improvements from T0 to T2 (*p* < 0.02) and from T0 to T4 (*p* < 0.001), with increases of approximately 21.8% and 37.4%, respectively. Rotation homolateral to the affected UT significantly increased in the TECAR group (*p* = 0.014), with post hoc analysis showing a significant improvement at T4 compared to T0 (approximately 14.2%). No significant changes were observed in the DN group for this parameter (*p* = 0.34). Finally, there was an approximate 16% significant increase in rotation contralateral to the affected UT from T0 to T4 in the TECAR group. In contrast, the DN group exhibited no statistically significant longitudinal changes in contralateral rotation during the course of the study (Table 5).

## 4. Discussion

The principal finding of our study suggests that TECAR therapy may be effective in attenuating pain and augmenting PPT in male subjects presenting with active MTrP within the UT. Notably, the analgesic effects associated with TECAR were more pronounced than those observed in the DN group following both treatment stages. Furthermore, TECAR therapy was associated with significant enhancements in specific cervical spine ROM, including flexion, contralateral rotation, and both contralateral and homolateral side bending during the second treatment stage, in contrast to the DN group, which exhibited less consistent ROM alterations in the same stage.

During stage I of the study, a significant decrease in pain intensity, as assessed by the NRS, was observed in men undergoing either therapy. Notably, the reduction in pain during T1 was significantly greater in the TECAR group. The potential analgesic effects of TECAR therapy have been already proven in numerous studies [24,25,26,27,28,42,43]. This response is often attributed to therapy-mediated heat (diathermy effect) production, which may lead to excessive vasodilatation and the release of pain mediators, such as bradykinin, serotonin, and prostaglandin [29]. Moreover, Tashiro et al. indicated increased oxyhemoglobin after TECAR application [44], which might directly affect MTrP’s metabolism stimulation and trigger the healing process. It has also been suggested that TECAR equalizes the resting potential of the cell membrane as a result of the high-frequency current flow. Two previous studies have reported comparable analgesic effect of TECAR therapy on active MTrP [42,43], providing a possible explanation for the pain reduction noted in the TECAR group. In addition, ∆NRS was statistically significantly greater in the TECAR group compared to the DN group during T3. This could potentially be attributed to the subjectivity of the NRS, the invasive nature of DN, and the post-needling soreness [45]. However, as post-treatment soreness was not formally quantified in the present study, future research should incorporate objective monitoring of this variable to better differentiate between therapeutic response and transient procedural discomfort. The application of DN is usually associated with post-needling-induced pain [46]. Contrary to TECAR, which is worth emphasizing, it is a non-invasive therapy. This distinction may explain why subjectively assessed pain reduction might have been greater in men undergoing TECAR therapy. In the DN group, we did not notice a significant decrease in pain intensity assessed with NRS at T3. Nevertheless, we observed at T1. DN-mediated pain mitigation is typically explained as a consequence of neurophysiological and chemical reactions [22,47,48]. Niddam et al. proposed that pain mediation after DN occurs through the brainstem’s periaqueductal grey substance, which proves that DN may activate enkephalinergic inhibitory dorsal horn interneurons [49]. Moreover, recent metanalyses [50,51] indicate that DN effectively decreases pain in immediate, short- and medium-term measurements compared to sham exposure groups.

Previous studies have demonstrated that PPT serves as an effective objective measure for quantifying pain, providing therapists with a reliable method to monitor treatment outcomes [52,53]. Both methods were associated with significant improvement in PPT during T1. Nevertheless, ∆PPT values did not exhibit significant differences between the groups, suggesting a potentially comparable short-term influence for both interventions. Subsequently, the TECAR group exhibited a more pronounced and statistically significant long-term enhancement of PPT, particularly evident in the statistically significant improvement observed during T3. This observation could be linked to changes in connective tissue structure, whereby TECAR’s influence on elasticity potentially reduces mechanical restrictions and improves muscle function [24].

Muscle strength has been rarely assessed in studies considering MTrPs in the UT. In our study, we have evaluated the UT strength with scapular elevation. The advantages of this concept are ease of execution and repeatability. Our results indicate that decreased muscle strength in UT with active MTrP is consistent with the available reports [1,3,8]. Considering the energy crisis hypothesis, muscle strength diminishment may result from the reduced availability of adenosine triphosphate, leading to sarcomere shortening [48]. A significant decrease in the UT strength on the unaffected side was noted at T1 in the TECAR therapy group, which was likely a consequence of the contralateral reflex. Therapy-mediated heat overproduction may activate the vascular system. Its activity is aimed at removing heat by transferring it to the opposite side of the body. Muscle strength measurements were taken directly after therapy, might have interfered with the vasomotor reflex on the unaffected side [54]. The explanation for the lack of improvement in muscle strength in the DN-mediated group might be explained by a potential decrease in UT activity as a result of needle puncture. De Meulemeester and Calders reported that a single DN session instantly led to a significant decrease in the UT surface electromyography activity (sEMG) [55].

During T0, all study participants exhibited a diminished ROM of the cervical spine, confirming the impact of MTrPs on its limitation [5,17]. Nevertheless, our study indicates that both TECAR and DN therapies facilitate an improvement in cervical spine ROM. This finding is consistent with the existing studies comparing the effectiveness of various modalities to DN in the treatment of MTrP [8,42,47,56,57].

Following T1 of the study, an improvement in cervical spine extension, side bending, and contralateral rotation to the affected UT was observed in either group. Moreover, during T1 of the study, there was an increase in the ROM for flexion in the TECAR and for side bending homolateral to the affected UT in the DN. Both treatment groups demonstrated effectiveness in reducing pain (as indicated by the NRS results). A decrease in pain may lead to the relaxation of tight band muscles in the cervical spine and shoulder girdle, directly translating to an increased ROM. Improvement in one ROM (e.g., extension) may indirectly influence other ranges (e.g., side bending or rotation) by potentially reducing compensatory movement patterns and restoring more optimal cervical spine biomechanics [58]. Future studies should aim to elucidate the specific biomechanical and neurophysiological mechanisms through which pain reduction in one cervical movement influences the ROM in other planes. This aspect was not a primary outcome of the current investigation.

At T3 of the study, the TECAR group demonstrated significant improvements in homolateral side bending, contralateral side bending, extension and homolateral rotation. This suggests that the therapeutic effects of TECAR on cervical ROM continue and potentially evolve with subsequent sessions. In contrast, the DN group did not exhibit statistically significant changes in any measured ROM at T3. While the DN therapy yielded significant improvements in cervical spine ROM at T1, encompassing various movements, at T3 data indicate a lack of sustained statistically significant ROM changes. The observed increase in rotation and side bending contralateral to the MTrP at T1 for both groups might be attributed to the relaxation of the UT fibers, potentially leading to improved concentric and eccentric activities. Furthermore, the decrease in pain intensity in both groups likely contributed to the increased ROM, as pain-free motion facilitates greater movement. Improvements in rotation and side bending may also involve synergic muscle activity, such as the levator scapulae and sternocleidomastoid muscles, which were not assessed in this study [14]. Moreover, both therapies have been suggested to enhance blood flow in the MTrP region, potentially reducing tissue hypoxia and secondary myosin-actin filament overlap, thereby decreasing muscle stiffness and improving ROM [22], which might have influenced the initial results.

Importantly, while individual groups showed significant ROM changes in specific movements, the comparative analysis of changes (∆ROM) between the TECAR and DN groups revealed a significant difference only in homolateral side bending, with the TECAR group exhibiting a greater increase.

Both TECAR and DN were associated with significantly reduced pain (NRS) across all stages (*p* < 0.05). Similarly, both groups showed significant increases in pressure PPT between stages (*p* < 0.05). Despite these observed beneficial changes in pain and PPT, the long-term effects on affected UT strength and the full cervical ROM appeared less consistent. TECAR was associated with improvement in flexion, contralateral side bending, and contralateral and homolateral rotation, while DN yielded improvements in extension and contralateral side bending. The lack of sustained changes in all ROM aspects and muscle strength at T4 might be attributed to participants’ unrestricted activity levels. Further research controlling activity is needed to better assess the long-term efficacy of both interventions.

Compared to previous studies, a key strength lies in the methodology of TECAR therapy, where we primarily focused only on the treatment of the MTrP area through the use of impulsive therapy. In contrast, other studies have targeted both the MTrP and adjacent muscles, such as the levator scapulae and paraspinal muscles, as well as the asymptomatic side [42,43]. Our focused approach may allow for a more precise isolation of the effects of TECAR on specific MTrPs pathophysiology.

### 4.1. Clinical Significance

The findings of this study provide clinically relevant insights for the management of MTrP in the UT. Both TECAR therapy and DN demonstrated the ability to significantly reduce pain intensity. TECAR therapy offers a non-invasive, thermal alternative for patients with “needle phobia” or those at risk of bleeding while providing comparable results to the mechanical stimulation of DN.

### 4.2. Study Limitations

The study has several limitations. In particular, the small number of participants and the stringent exclusion criteria may reduce the extent to which the findings can be generalized. In addition, the limited sample size may have affected statistical power, and consequently, statistically significant differences should be interpreted with caution. Second, the present study did not include a sham control group or non-treated individuals; therefore, it may impact the interpretation of causal effects. Third, the application of only two therapeutic sessions may limit broader clinical interpretation regarding long-term outcomes; however, this protocol was specifically designed to evaluate immediate physiological responses and is supported by existing literature demonstrating significant clinical improvements following a two-session intervention [59]. Additionally, the same researcher administered the treatment, potentially leading to measurement bias. Nevertheless, the evaluator was a highly trained physiotherapist. Future studies should ensure that the individual conducting assessments is not involved in delivering the intervention.

### 4.3. Recommendations

Based on the current findings, future studies should consider several directions. First, multimodal protocols should be investigated, particularly the potential synergistic effects of combining TECAR or DN with progressive resistance training or postural re-education. Second, multi-center randomized controlled trials with larger sample sizes should be conducted to increase statistical power and improve the generalizability of the findings. Third, future research could incorporate biochemical analyses, such as microdialysis or ultrasound elastography, to objectively assess changes in the biochemical environment and tissue stiffness of myofascial trigger points following the intervention. Finally, the efficacy of these treatments should be examined in specific occupational populations, such as office workers or overhead athletes, who are particularly prone to chronic upper trapezius dysfunction.

## 5. Conclusions

TECAR therapy appears to provide both immediate and longer-term analgesic effects in the treatment of active MTrPs in the UT, although its impact on cervical ROM seems to be relatively limited compared with DN. Given these findings, TECAR therapy may represent a potentially useful, though less commonly applied, option for the management of MTrPs.

## Figures and Tables

**Table 1:** Characteristics of the participants.

		TECAR	DN	*p* Value
**Age [years] (M ± SD)**		34.94 ± 6.63	36.33 ± 4.29	0.53
**Body weight [kg] (M ± SD)**		83.37 ± 13.13	93.50 ± 6.93	0.02
**Body mass index [kg/m^2^] (M ± SD)**		25.63 ± 3.1	27.15 ± 2.01	0.15
**Body fat [%] (M ± SD)**		23.10 ± 4.66	22.55 ± 4.63	0.76
**MTrP location**	Right	11	9	0.56
	Left	6	3	

Legend: TECAR—capacitive and resistive energy transfer; DN—dry needling.

**Table 2:** PPT [kg/cm^2^] and NRS [points] in TECAR and DN groups.

**Pressure Pain Threshold [kg/cm^2^] (M ± SD)**					
	**Stage 1**			**Stage 2**	
	**DN**	**TECAR**		**DN**	**TECAR**
T0	8.34 ± 1.64	6.52 ± 2.35	T2	11.32 ± 2.07	9.18 ± 4.06
T1	10.40 ± 2.42	9.49 ± 3.45	T3	12.19 ± 2.83	10.64 ± 3.49
*p*^1^ value	0.01	0.001	*p*^1^ value	0.38	0.001
∆	−2.05 ± 2.41	−2.98 ± 2.54	∆	−0.88 ± 2.82	−1.44 ± 2.18
*p*^2^ value	0.36		*p*^2^ value	0.39	
**Numeric rating scale [points] (M ± SD)**					
	**Stage 1**			**Stage 2**	
	**DN**	**TECAR**		**DN**	**TECAR**
T0	4.33 ± 1.87	5.82 ± 1.81	T2	1.92 ± 1.38	2.76 ± 2.14
T1	3.50 ± 2.24	1.79 ± 1.85	T3	2.17 ± 1.64	0.62 ± 0.82
*p*^1^ value	0.013	0.001	*p*^1^ value	1.00	0.001
∆	0.83 ± 1.99	4.03 ± 1.75	∆	−0.25 ± 1.86	2.15 ± 1.84
*p*^2^ value	0.001		*p*^2^ value	0.002	

Legend: *p*^1^ value—intragroup comparison; *p*^2^ value—intergroup comparison. T0—baseline measurement; T1—measurement immediately following the first session; T2—measurement before second session; T3—measurement immediately following the second session.

**Table 3:** The muscle strength of UT [kG] at each stage of the study in TECAR and DN groups.

**Strength of the Affected UT [kG] (M ± SD)**					
	**Stage 1**			**Stage 2**	
	**DN**	**TECAR**		**DN**	**TECAR**
T0	31.56 ± 10.94	29.43 ± 8.38	T2	34.53 ± 12.80	29.20 ± 8.95
T1	31.29 ± 7.34	27.85 ± 7.32	T3	32.22 ± 12.32	27.89 ± 8.65
*p*^1^ value	0.86	0.12	*p*^1^ value	0.22	1.00
∆	0.28 ± 5.44	1.57 ± 3.99	∆	2.30 ± 2.93	1.31 ± 4.61
*p*^2^ value	0.61		*p*^2^ value	0.47	
**Strength of the unaffected UT [kG] (M ± SD)**					
	**Stage 1**			**Stage 2**	
	**DN**	**TECAR**		**DN**	**TECAR**
T0	37.23 ± 10.88	33.61 ± 9.37	T2	38.99 ± 13.40	31.22 ± 9.28
T1	37.15 ± 15.29	30.13 ± 9.08	T3	36.50 ± 12.69	29.47 ± 9.18
*p*^1^ value	0.97	0.01	*p*^1^ value	0.15	0.08
∆	0.07 ± 5.91	3.49 ± 3.71	∆	2.48 ± 4.53	1.75 ± 2.95
*p*^2^ value	0.03		*p*^2^ value	0.66	

Legend: *p*^1^ value—intragroup comparison; *p*^2^ value—intergroup comparison. T0—baseline measurement; T1—measurement immediately following the first session; T2—measurement before second session; T3—measurement immediately following the second session.

**Table 4:** Cervical spine ROM in TECAR and DN groups.

**Flexion [°] (M ± SD)**					
	**Stage 1**			**Stage 2**	
	**DN**	**TECAR**		**DN**	**TECAR**
T0	32.92 ± 8.91	32.35 ± 8.50	T2	35.83 ± 7.93	37.35 ± 6.87
T1	37.50 ± 6.57	39.12 ± 5.07	T3	37.08 ± 7.22	39.71 ± 4.83
*p*^1^ value	0.07	0.04	*p*^1^ value	1	0.13
∆	−4.58 ± 6.56	−6.76 ± 6.83	∆	−1.25 ± 9.80	−2.35 ± 4.72
*p*^2^ value	0.39		*p*^2^ value	0.58	
**Extension [°] (M ± SD)**					
	**Stage 1**			**Stage 2**	
	**DN**	**TECAR**		**DN**	**TECAR**
T0	35.83 ± 8.75	34.71 ± 11.25	T2	44.17 ± 6.34	38.53 ± 8.80
T1	41.25 ± 7.42	39.41 ± 7.68	T3	44.58 ± 6.20	40.59 ± 6.82
*p*^1^ value	0.04	0.04	*p*^1^ value	0.82	0.04
∆	−5.42 ± 6.89	−4.71 ± 9.76	∆	−0.42 ± 1.44	−2.06 ± 3.09
*p*^2^ value	0.98		*p*^2^ value	0.22	
**Side bending homolateral to affected UT [°] (M ± SD)**					
	**Stage 1**			**Stage 2**	
	**DN**	**TECAR**		**DN**	**TECAR**
T0	35.42 ± 6.56	35.88 ± 6.90	T2	39.58 ± 7.22	35.29 ± 8.38
T1	41.25 ± 5.28	37.06 ± 7.30	T3	38.33 ± 7.49	40.59 ± 4.29
*p*^1^ value	0.03	0.63	*p*^1^ value	0.48	0.008
∆	−5.83 ± 6.34	−1.18 ± 7.81	∆	1.25 ± 3.11	−5.29 ± 6.24
*p*^2^ value	0.1		*p*^2^ value	0.006	
**Side bending contralateral to affected UT [°] (M ± SD)**					
	**Stage 1**			**Stage 2**	
	**DN**	**TECAR**		**DN**	**TECAR**
T0	34.16 ± 5.57	29.71 ± 6.24	T2	38.75 ± 3.77	36.18 ± 4.52
T1	40.00 ± 3.69	36.59 ± 8.24	T3	40.00 ± 3.02	40.59 ± 6.09
*p*^1^ value	0.006	0.009	*p*^1^ value	0.48	0.02
∆	−5.83 ± 6.69	−6.88 ± 7.47	∆	−1.25 ± 3.11	−4.41 ± 4.96
*p*^2^ value	0.7		*p*^2^ value	0.06	
**Rotation homolateral to affected UT [°] (M ± SD)**					
	**Stage 1**			**Stage 2**	
	**DN**	**TECAR**		**DN**	**TECAR**
T0	61.67 ± 5.82	60.59 ± 7.68	T2	64.58 ± 6.56	64.71 ± 6.95
T1	68.13 ± 5.3	67.94 ± 9.53	T3	66.67 ± 4.92	69.12 ± 6.90
*p*^1^ value	0.18	0.08	*p*^1^ value	0.48	0.02
∆	−3.75 ± 8.56	−7.35 ± 10.91	∆	−2.08 ± 5.82	−4.41 ± 5.27
*p*^2^ value	0.35		*p*^2^ value	0.12	
**Rotation contralateral to affected UT [°] (M ± SD)**					
	**Stage 1**			**Stage 2**	
	**DN**	**TECAR**		**DN**	**TECAR**
T0	60.42 ± 9.88	58.53 ± 10.86	T2	65.42 ± 6.56	64.41 ± 7.05
T1	67.08 ± 5.42	66.18 ± 4.52	T3	63.33 ± 7.49	66.76 ± 5.04
*p*^1^ value	0.045	0.02	*p*^1^ value	0.48	0.22
∆	−6.67 ± 10.08	−7.65 ± 11.47	∆	2.08 ± 5.82	−2.35 ± 6.35
*p*^2^ value	0.81		*p*^2^ value	0.11	

Legend: *p*^1^ value—intragroup comparison; *p*^2^ value—intergroup comparison. T0—baseline measurement; T1—measurement immediately following the first session; T2—measurement before second session; T3—measurement immediately following the second session.

**Table 5:** Consecutive cervical spine range of motion measurements in TECAR and DN groups.

**Flexion [°] (M ± SD)**	**T0**	**T2**	**T4**	***p* value**	**post hoc**
DN	32.91 ± 8.91	35.83 ± 7.93	37.92 ± 7.82	0.34	-
TECAR	32.35 ± 8.50	37.35 ± 6.87	39.17 ± 6.34	0.04	I < III (0.048)
**Extension [°] (M ± SD)**	**T0**	**T2**	**T4**	***p* value**	
DN	35.83 ± 8.75	44.17 ± 6.34	44.58 ± 6.20	0.007	I < II (0.02) I < III (0.01)
TECAR	34.71 ± 11.25	38.53 ± 8.80	40.00 ± 6.40	0.28	-
**Side bending homolateral to affected UT [°] (M ± SD)**	**T0**	**T2**	**T4**	***p* value**	
DN	35.42 ± 6.56	39.58 ± 7.22	40.00 ± 7.07	0.21	-
TECAR	35.88 ± 6.90	35.29 ± 8.38	39.17 ± 5.57	0.33	**-**
**Side bending contralateral to affected UT [°] (M ± SD)**	**T0**	**T2**	**T4**	***p* value**	
DN	34.17 ± 5.57	38.75 ± 3.77	40.00 ± 3.02	0.006	I < II (0.03) I < III (0.006)
TECAR	29.71 ± 6.24	36.18 ± 4.52	40.83 ± 4.17	0.01	I < II (0.02) I < III (0.001)
**Rotation homolateral to affected UT [°] (M ± SD)**	**T0**	**T2**	**T4**	***p* value**	
DN	61.67 ± 7.49	64.58 ± 6.56	65.42 ± 4.98	0.34	-
TECAR	60.59 ± 7.68	64.71 ± 6.95	69.17 ± 7.64	0.014	I < III (0.01)
**Rotation contralateral to affected UT [°] (M ± SD)**	**T0**	**T2**	**T4**	***p* value**	
DN	60.42 ± 9.88	65.42 ± 6.56	66.25 ± 4.33	0.27	-
TECAR	58.53 ± 10.86	64.41 ± 7.05	67.92 ± 3.34	0.02	I < III (0.03)

## Data Availability

The original contributions presented in this study are included in the article. Further inquiries can be directed to the corresponding author.
